# Predictive Model for Oral Status in Elderly People in a Taiwanese Nursing Home Using a High-Protein Black Soybean Koji Food

**DOI:** 10.3389/fnut.2022.814315

**Published:** 2022-04-14

**Authors:** Kai-Wei Liao, Wen-Liang Lo, Chien-Yeh Hsu, Wan-Chun Chiu, Chieh-Hsi Wu, Yi-Wen Chen, Po-Chi Hsu, Hui-Yu Huang

**Affiliations:** ^1^School of Food Safety, College of Nutrition, Taipei Medical University, Taipei, Taiwan; ^2^Department of Dentistry, National Yang Ming Chiao Tung University, Taipei, Taiwan; ^3^Department of Stomatology, Division of Oral and Maxillofacial Surgery, Taipei Veterans General Hospital, Taipei, Taiwan; ^4^Department of Information Management, National Taipei University of Nursing and Health Science, Taipei, Taiwan; ^5^School of Nutrition and Health Sciences, Taipei Medical University, Taipei, Taiwan; ^6^School of Pharmacy, Taipei Medical University, Taipei, Taiwan; ^7^Graduate Institute of Metabolism and Obesity Sciences, Taipei Medical University, Taipei, Taiwan

**Keywords:** elderly, nursing home, texture classification, high-protein black soybean koji product, oral status

## Abstract

With increased age, the appetite, chewing, swallowing, and digestive ability gradually decrease. Previous studies have shown that poor oral health is associated with an inadequate intake of macro and micronutrients and malnutrition. Therefore, improving the diet of elderly people and promoting nutrient absorption will help to improve the quality of life for elderly people. However, few studies have predicted their oral ability based on different food textures and other factors. The purpose of this study was to explore the correlation between oral assessment and texture parameters of high-protein black soybean koji products in elderly people in a nursing home. We used cross-sectional study design for seventy-nine residents aged 65 years and older were recruited. Three different texture of cookies, including normal cookie hardness (1.4 × 10^5^ N/m^2^), minced cookie hardness (4.4 × 10^4^ N/m^2^), and pureed cookie hardness (1.4 × 10^4^ N/m^2^) were provided to participants to test the oral status. An oral assessment scale was used by a dentist to evaluate the oral status of the elderly participants. Different cookie textures showed a significant positive correlation with pronunciation (*r* = 0.237, *p* < 0.05), face (*r* = 0.371, *p* < 0.01), tongue (*r* = 0.362, *p* < 0.01), pharynx (*r* = 0.256, *p* < 0.05), swallowing (*r* = 0.272, *p* < 0.05), breathing (*r* = 0.315, *p* < 0.01), and the total oral score (*r* = 0.339, *p* < 0.01). We also used the high-protein black soybean koji products combined with elderly people’s comprehensions in a predictive model that had a moderately high correlation to predict the oral status in the elderly group (*r* = 0.612). We concluded that the high-protein black soybean koji product was associated with the oral ability of elderly people in a nursing home in Taiwan. Our findings indicated that elderly people could immediately understand the correct food texture.

## Introduction

The world’s population is rapidly aging, and the proportion of elderly people is estimated to be approximately 12 to 22% worldwide (1, 2). In Taiwan, the elderly population percentage is likely to reach 39.3% by 2060, and this aging population percentage will be the second highest in the world (3). Oral health status is an important factor for nutrition in elderly people. Poor oral health and oral function may cause nutritional deficiencies or changes in food preferences when a tooth is lost (4). Furthermore, oral performance, especially mastication, which is closely related to the quality of life, is essential to maintain activities of daily living for elderly people (5).

Malnutrition is a major problem in nursing homes, and it is an issue for up to 85% of the elderly residents (6, 7). Nursing homes face the challenge of having to ensure adequate nutritional care, considering differing concurrent requirements and wishes of their residents (8). A previous study has found that oral residence time was significantly positively correlated with the number of chews, liking, and difficulty perceived (9). Correctly identifying the diet texture is important for elderly people to support adequate nutrition intake. Because many residents are not able to eat food with a regular consistency due to chewing or swallowing problems, different degrees of texture modification are required to ensure a safe swallowing process as well as to maintain and improve the ability to eat. Some studies have reported tools for measuring the oral status in elderly groups (7, 10, 11). However, it takes a long time to ensure that food textures are suitable for elderly nursing home residents.

Oral abilities (e.g., swallowing and chewing difficulties) are often noted as part of the many factors that cause malnutrition in long-term care facilities (12). However, little research has been conducted on how to predict oral ability based on different food textures and other demographics, while preserving the correct food texture and sufficient nutritional intake to minimize the prevalence of undernutrition due to the potential effect of this food manipulation.

In the current study, we developed a high-protein black soybean koji product and established an oral assessment scale for elderly people in Taiwan. We aimed to explore the correlation between oral assessment scores, textures of high-protein black soybean koji products, and feeding styles of elderly people in a nursing home. In addition, we want to find a way to quickly determine the oral ability of elderly people to find the appropriate food texture for their meal.

## Materials and Methods

### Ethics Statement

This study was approved by the University of Taipei Institutional Review Board (IRB-2017-015) in Taiwan. Written informed consent from each participant was obtained before study enrollment.

### Subject Recruitment

A cross-sectional study was conducted. Subjects were recruited from an elderly long-term care facility in the SanXia Qingfu Nursing Home in Xinbei City, Taiwan between 2017 and 2018. All participants received an oral assessment by the same dentist to evaluate their oral status and tasted cookie products with different textures. Three different textures of high-protein black soybean koji products are provided to participants. They will eat the different textures of cookies, and choose the most willing to eat. Eighty-four residents aged 65 years and older were recruited and excluded the participants who with nasogastric tube care.

### Oral Status Assessment Scale

We modified the procedure that was described in previous oral assessment studies (13–16) and determined Taiwan’s elderly group oral status assessment. Detailed oral assessment scale was presented in Supplementary Table 1. The oral status assessment items were included three important factors, namely chewing, swallowing, and other factors. Chewing factors were included the phonation (including the pronunciation of pa, ta, ka, and ra, full scores: 12), facial movement (including lip closure, smiling, cheek sucking, and cheek blowing, full scores: 12), and gnathic-glossal movement (including mouth opening, tongue thrust, tongue tip up, tongue base up, tongue swing, tongue resistance, and tongue sensation, full scores: 24). Swallowing factors included neck movement (including flexion, extension, waving, and symptom, full scores: 13), larynx (including uvula movement, uvula deviation, rhinorrhea when expiration, soft palate sensation, soft palate reflex, and larynx reflex, full scores: 16), and swallowing function (including larynx up, Repetitive Saliva Swallowing Test (RSST), exhalation sound when rest, swallowing sound, and post-swallowing exhalation sound, full scores: 17). Other factors included respiratory condition (including diaphragmatic breathing, random cough, and blowing, full scores: 9), oral cavity (oral hygiene, peel off adhesion, fur adhesion, and mouth dryness, full scores: 12), consciousness, understanding, movement, and intake condition. We summarized the total oral score (the highest scores: 115), which was determined by adding together the above factor’s scores.

### High-Protein Black Soybean Koji Products

**FIGURE 1:**
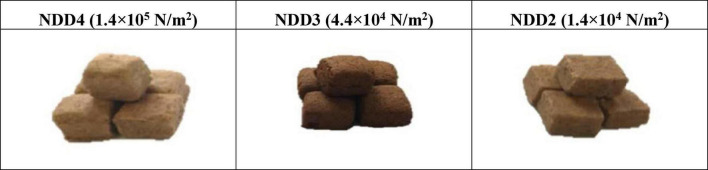
Different textures of cookie.

Based on the standards of the Universal Design Foods (UDF), the International Dysphagia Diet Standardisation Initiative (IDDSI), and the Taiwan Food Industry Research and Development Institute (FIRDI), we developed a high-protein black soybean koji product for elderly people using three kinds of cookies. We used the texture profile analysis to identify the three types of cookies, including a normal cookie hardness (1.4 × 10^5^ N/m^2^, NND4), minced cookie hardness (4.4 × 10^4^ N/m^2^, NND3), and pureed cookie hardness (1.4 × 10^4^ N/m^2^, NND2). The content of normal cookie (NND4) made with plain flour, unsalted butter, erythritol, egg, black soybean koji powder, and water, and the processes were oven at 180°C and bake at 160°C for 8 min. For minced cookie (NND3) made with plain flour, unsalted butter, erythritol, egg, black soybean koji powder, water, salt, and baking soda. The processes were preheated in the oven at 180°C, bake at 180°C for 5 min, then bake at 140°C for 5∼10 min. For pureed cookie (NDD2) made with yolk, potato starch, black soybean koji powder, erythritol, and vegetable oil. The processes were preheated oven to 170°C and bake for 8–12 min. The figures of cookies as shown in Figure 1.

### Demographic Information

One well-trained research assistant collected demographic information from all participants, including age, height, weight, and physical activity (walking, wheelchair users, or bedridden). These data were given by the participants.

### Statistical Analysis

The demographics of the participants are presented. The distribution of calorie intake and the scores for each oral status, physical activity, and cookie texture are presented as the median (range). The Chi-square or Fisher’s exact test was used to categorize variables, and the Kruskal-Wallis test was used to evaluate differences in continuous variables. The Spearman correlation was applied to determine the correlation between the cookie texture and oral assessments. Stepwise multiple regression was applied to test for putative factors that influenced oral status. All analyses were performed using SPSS version 19.0 (version 19.0, IBM Corporation, Armonk, NY, United States). Statistical significance was defined as a *p* < 0.05.

## Results

### Study Population

Table 1 lists the demographic characteristics of the elderly participants by pureed (*n* = 11), minced (*n* = 21), and usual diet (*n* = 47) groups. There was a significantly different age (*p* = 0.010) and BMI (*p* = 0.019) between the three groups. Participants in the minced group were older than those in the other groups, while BMI increased by dietary type from pureed, which was the lowest, to minced to the usual diet, which was the largest. The median scores for phonation, facial movement, gnathic-glossal movement, neck movement, larynx, swallowing function, respiratory function, oral cavity, and total oral scores in the pureed/minced/usual diet groups were 10/11/12, 10/11/12, 18/17/20, 9/13/13, 13/13/16, 4/9/16, 6/6/7, 9/10/11, and 83/87/102, respectively. The usual diet group had the highest scores for all items compared to the other groups. For comprehension, we found that the pureed group had the highest percentage of worse comprehensive and the usual diet group had the highest percentage of good comprehensive (*p* = 0.046). We also used high-protein black soybean koji cookies with different textures to predict the oral ability of the elderly participants. We found that the largest number of elderly participants who chose the NND2, NND3, and NND4 cookies were in the pureed (46.7%), minced (38.1%), and usual diet (77.1%) groups, respectively (Table 1). In the current study, we found that over 45% of the elderly participants who received pureed food could bite the normal and minced cookies. This represents a difficulty for nursing homes to determine the correct types of food for the elderly residents.

**TABLE 1:** Characteristics of subjects in a nursing house (*N* = 79).

	Pureed (*n* = 11)	Minced (*n* = 21)	Usual diet (*n* = 47)	
	Median (range), n (%)	Median (range), n (%)	Median (range), n (%)	*p*-value^a^
Gender (male/female)	4 (36.4)/7 (63.6)	6 (28.6)/16 (71.4)	18 (38.3)/29 (61.7)	0.501
Age (years)	83 (71–94)	86 (70–95)	79 (66–99)	0.010*
Height (cm)	158 (148–170)	150 (141–168)	157 (137–173)	0.014*
Weight (kg)	53 (40–66)	50 (38–102)	59 (37–76)	0.015*
BMI (kg/m^2^)	19.7 (17.9–25.5)	23.3 (17.7–39.8)	24.0 (16.6–32.5)	0.019*
**Scores**				
Pronunciation	10 (0–12)	11 (0–12)	12 (0–12)	0.007**
Face	10 (0–12)	11 (6–12)	12 (0–12)	<0.001**
Tongue	18 (8–21)	17 (0–22)	20 (0–23)	0.042*
Neck	9 (0–13)	13 (4–13)	13 (0–13)	0.006**
Pharynx	13 (7–16)	13 (8–16)	16 (5–16)	0.011*
Swallowing	4 (0–17)	9 (0–17)	16 (0–17)	<0.001**
Breathing	6 (0–9)	6 (0–9)	7 (0–9)	0.015*
Oral cavity	9 (5–12)	10 (8–12)	11 (2–12)	0.380
Total oral scores	83 (30–109)	87 (51–104)	102 (21–113)	<0.001**
**Physical activity**				0.721
Walking	3 (27.3)	9 (42.9)	14 (29.8)	
Wheelchair users	6 (54.5)	9 (42.9)	28 (59.6)	
Bedridden	2 (18.2)	3 (14.2)	5 (10.6)	
**Comprehension**				0.046*
Good	3 (27.3)	14 (66.7)	34 (72.3)	
Bad	3 (27.3)	3 (14.3)	6 (12.8)	
Worse	5 (45.4)	4 (19.0)	7 (14.9)	
**Cookie texture**				<0.001**
NND4	3 (27.3)	6 (28.6)	37 (78.8)	
NND3	2 (18.2)	8 (38.1)	5 (10.6)	
NND2	6 (54.5)	7 (33.3)	5 (10.6)	

^a^Comparison of three groups using Chi-square, Fisher’s exact test or Kruskal–Wallis test, *p < 0.05, **p < 0.01.

### Spearman Correlations Between Cookie Texture, Nursing Home Feeding, and Oral Ability Assessment

**FIGURE 2:**
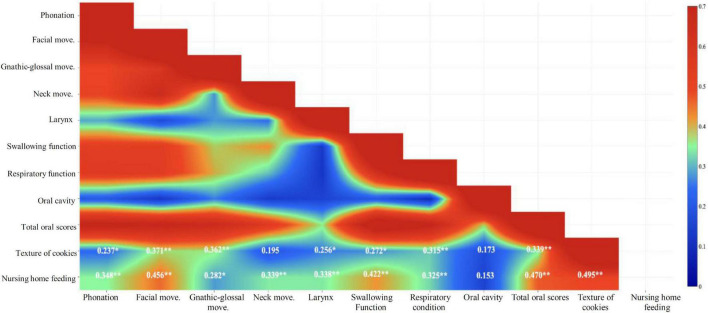
Spearman correlations between cookie texture and nursing home feeding and oral assessments (*p < 0.05, **p < 0.01).

The cookie texture showed a significant positive correlation with phonation (*r* = 0.237, *p* < 0.05), facial movement (*r* = 0.371, *p* < 0.01), gnathic-glossal movement (*r* = 0.362, *p* < 0.01), larynx (*r* = 0.256, *p* < 0.05), swallowing function (*r* = 0.272, *p* < 0.05), respiratory function (*r* = 0.315, *p* < 0.01), and total oral scores (*r* = 0.339, *p* < 0.01) (Figure 2 and Supplementary Table 2).

**FIGURE 3:**
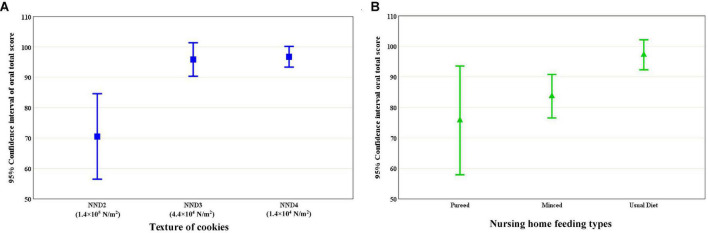
Cookie texture **(A)** and nursing home feeding type **(B)** according to the total score for the oral assessments (p for trend ≤ 0.001).

The elderly participants who chose NND3 and NND4 had significantly higher total oral scores than those who chose NND2. However, total oral score for the elderly participants who received the pureed and minced cookies was not significantly different, and only the usual diet group had a significantly higher total oral score compared to the other groups (Figure 3).

### Associations Between Black Soybean Cookies, Nursing Home Feeding, and Total Oral Scores

Positive correlations with using different models and total oral scores were observed in this study. We used stepwise regression to determine the best regression model (the potential confounding facts including age, gender, BMI, physical activity, and comprehension for the correlation between oral cookie textures and nursing home feeding styles. The results showed that cookie texture combined with elderly participant comprehension had a moderately strong correlation (*r* = 0.612), whereas the nursing home feeding style combined with physical activity and participant comprehension had a weaker correlation (*r* = 0.544) (Table 2).

**TABLE 2:** Stepwise regression model of total oral scores and cookie texture in an elderly population (*N* = 79).

	Total oral scores			
	Beta	*p*-value	Beta	*p*-value
Constant	37.663	<0.001	39.763	<0.001
Physical activity			6.578	0.035
Comprehension	6.071	<0.001	3.819	0.038
Texture of cookies	11.667	<0.001		
Nursing home feeding styles			9.914	<0.001
R	0.612		0.544	
R^2^	0.374		0.296	

## Discussion

To the best of our knowledge, this is the first study to assess oral health and consider best practices for different food textures in the elderly population in a Taiwanese nursing home. We found a moderately strong correlation (*r* = 0.495, *p* < 0.01) between the textures of our products (high-protein black soybean koji products) and feeding textures from the nursing home. We also assessed the different status of oral ability scores to understand oral health. Furthermore, we used a predictive model to predict the oral status in the elderly participants and used the high-protein black soybean koji products to represent a good prediction for elderly people in Taiwan.

Our results represent a difficulty for nursing homes to determine the correct types of food for the elderly residents. Problems often occur during interactions between the elderly residents and care staff in meal situations, where refusal to eat or declining assistance from care staff can lead to negative interactions and inappropriate food textures. In a review study, the high prevalence rate of swallowing (7%–68%) and chewing (11%–57%) problems in nursing homes was based on 17 studies (17). In our study, six (7.6%) elderly participants who ate pureed food chose the NND2 cookie. Seven (33.3%) and five (10.6%) elderly participants who received the minced and usual diet, respectively, at the nursing home chose the NND2 cookie. An immediate effect of eating inappropriately prepared food is reduced food intake, which increases the risk of malnutrition (18).

This limited study used different food textures combined with a predictive model to predict the oral status in the elderly participants. We considered many factors, including demographic characteristics (including age, gender, and BMI), physical activity, and comprehension because these factors are associated with oral status and oral health interest, and based on previously published studies, we developed a predictive model. We used a stepwise regression model to determine the best predictive model, and the best prediction was obtained when we used food texture combined with the elderly participants’ comprehension. The results showed a strong correlation coefficient (*r* = 0.612) between our high-protein black soybean koji products and the elderly participants’ oral status. Although the nursing home feeding style prediction model had a similar predictive power (*r* = 0.544), one more factor (physical activity) should be considered. Additionally, a long time should be taken to determine the nursing home feeding style to make sure that the appropriate feeding style is used. Our products and the predictive model provide an immediate way to determine the appropriate feeding style for elderly people.

We used the oral status assessment scale to evaluate oral health in elderly people in a nursing home. Eight categories were used to assess the oral health, and the item scores from each of these categories was added together as the total oral score that represented the elderly oral status. We found that the NND2 cookie was chosen by participants with significantly lower total oral scores compared with the other cookie groups. Cookies with different textures can be used to assess the oral status. However, nursing home feeding styles were not significantly different between each group, which means that the correlation between nursing home feeding style and oral status could not be determined. Thus, more precise oral status assessments are warranted.

The main strength of this study was its use of high-protein black soybean koji products as a predictor to understand the oral ability of elderly people in a nursing home. The high-protein black soybean koji products can be used by a nursing home to evaluate elderly residents and determine the best texture of food that they should receive. The participants’ oral ability was also assessed by a dentist, and we found a positive correlation between the oral assessment scores and the texture of high-protein black soybean koji products. This study also had some limitations. First, the sample size in our study groups was relatively small, and thus, studies using a large sample size are required to confirm our findings. Second, tooth health status in the elderly participants should be considered, which requires further research.

## Conclusion

A high-protein black soybean koji product was associated with oral ability of elderly nursing home residents in Taiwan. The predictive model showed good predictive ability for oral status in elderly residents of a Taiwanese nursing home. Our findings indicate that elderly people could quickly be provided with the appropriate food texture.

## Data Availability Statement

The original contributions presented in the study are included in the article/Supplementary Material, further inquiries can be directed to the corresponding author.

## Ethics Statement

The studies involving human participants were reviewed and approved by University of Taipei Institutional Review Board (IRB-2017-015) in Taiwan. The patients/participants provided their written informed consent to participate in this study.

## Author Contributions

W-LL and H-YH: conceptualization. W-LL, W-CC, and H-YH: methodology. K-WL, C-YH, and Y-WC: formal analysis. Y-WC and P-CH: investigation. H-YH, W-LL, and C-HW: resources. K-WL, W-LL, and H-YH: writing—original draft preparation. K-WL, W-LL, and H-YH: writing—review and editing. W-LL and H-YH: visualization. H-YH: supervision, project administration, funding acquisition, and data curation. All authors have read and agreed to the published version of the manuscript.

## Conflict of Interest

The authors declare that the research was conducted in the absence of any commercial or financial relationships that could be construed as a potential conflict of interest.

## Publisher’s Note

All claims expressed in this article are solely those of the authors and do not necessarily represent those of their affiliated organizations, or those of the publisher, the editors and the reviewers. Any product that may be evaluated in this article, or claim that may be made by its manufacturer, is not guaranteed or endorsed by the publisher.
