# Supplementary material for: Predictive Model for Oral Status in Elderly People in a Taiwanese Nursing Home Using a High-Protein Black Soybean Koji Food
Source: Front Nutr. 2022 Apr 14;9:814315. doi: 10.3389/fnut.2022.814315 (PMC9047994; doi:10.3389/fnut.2022.814315)
Supplement: Supplementary file 1 [file Data_Sheet_1.docx]

Supplementary Material

Table S1. Oral assessment scale

| **Factors** | | **Items** | **scores** |
| --- | --- | --- | --- |
| Chewing factors | Phonation | pa | 3：good, 2： poor, 1： bad, 0：uncooperative |
|  |  | ta | 3：good, 2： poor, 1： bad, 0：uncooperative |
|  |  | ka | 3：good, 2： poor, 1： bad, 0：uncooperative |
|  |  | ra | 3：good, 2： poor, 1： bad, 0：uncooperative |
|  | Facial movement | lip closure | 3：good, 2： poor, 1： bad, 0：uncooperative |
|  |  | smiling | 3：good, 2： poor, 1： bad, 0：uncooperative |
|  |  | cheek sucking | 3：good, 2： poor, 1： bad, 0：uncooperative |
|  |  | cheek blowing | 3：good, 2： poor, 1： bad, 0：uncooperative |
|  | Gnathic-glossal movement | mouth opening | 6：Above 60mm, 5：50-59, 4：40-49, 3：30-39, 2：20-29, 1：less than 19, 0：uncooperative |
|  |  | tongue thrust | 3：good, 2： poor, 1： bad, 0：uncooperative |
|  |  | tongue tip up | 3：good, 2： poor, 1： bad, 0：uncooperative |
|  |  | tongue base up | 3：good, 2： poor, 1： bad, 0：uncooperative |
|  |  | tongue swing | 3：good, 2： poor, 1： bad, 0：uncooperative |
|  |  | tongue resistance | 3：good, 2： poor, 1： bad, 0：uncooperative |
| Swallowing factors | Neck movement | flexion | 3：good, 2： poor, 1： bad, 0：uncooperative |
|  |  | extension | 3：good, 2： poor, 1： bad, 0：uncooperative |
|  |  | waving | 3：good, 2： poor, 1： bad, 0：uncooperative |
|  |  | symptom | 4：none, 3： numbness, 2： contraction, 1： pain, 0： uncooperative |
|  | Larynx | uvula movement | 3：good, 2： poor, 1： bad, 0：uncooperative |
|  |  | uvula deviation | 2： no, 1： yes, 0：uncooperative |
|  |  | rhinorrhea when expiration | 2： no, 1： yes, 0：uncooperative |
|  |  | soft palate sensation | 3：yes, 2： low, 1： no, 0：uncooperative |
|  |  | soft palate reflex | 3：yes, 2： low, 1： no, 0：uncooperative |
|  |  | larynx reflex | 3：yes, 2： low, 1： no, 0：uncooperative |
|  | Swallowing function | larynx up | 4：none, 3： numbness, 2： contraction,1： pain, 0： uncooperative |
|  |  | RSST Repetitive Saliva Swallowing Test | 2：>3 times, 1：<3 times, 0：did not perform |
|  |  | exhalation sound when rest | 4：clear, 3：wet sound, 2：liquid vibration sound, 1：garling sound, 0：uncooperative |
|  |  | swallowing sound | 4：no problem, 3：long, 2：weak, 1：multiple, 0：uncooperative |
|  |  | post-swallowing exhalation sound | 4：clear, 3：wet sound, 2：liquid vibration sound, 1：garling sound, 0： uncooperative |
| Other factors | Respiratory condition | diaphragmatic breathing | 3：good, 2：poor, 1：bad, 0: uncooperative |
|  |  | random cough | 3：good, 2：poor, 1：bad, 0: uncooperative |
|  |  | blowing | 3：good, 2：poor, 1：bad, 0: uncooperative |
|  | Oral cavity | oral hygiene | 3: good, 2：mediocre, 3：poor, 4：uncooperative |
|  |  | peel off adhesion | 3：no, 2：few, 1：many, 0：uncooperative |
|  |  | fur adhesion | 3：no, 2：few, 1：many, 0：uncooperative |
|  |  | mouth dryness | 3：no, 2：weak, 1：strong, 0：uncooperative |

Table S2. Spearman correlation coefficients of oral assessment and texture of cookies and nursing home feeding (N= 79).

|  | Pronunciation | Face | Tongue | Neck | Pharynx | Swallow | Breath | Oral cavity | Total oral scores | Texture of cookies | Nursing home feeding |
| --- | --- | --- | --- | --- | --- | --- | --- | --- | --- | --- | --- |
| Pronunciation | 1 |  |  |  |  |  |  |  |  |  |  |
| Face | 0.689** | 1 |  |  |  |  |  |  |  |  |  |
| Tongue | 0.495** | 0.573** | 1 |  |  |  |  |  |  |  |  |
| Neck | 0.505** | 0.671** | 0.275* | 1 |  |  |  |  |  |  |  |
| Pharynx | 0.289** | 0.170 | 0.277** | 0.244* | 1 |  |  |  |  |  |  |
| Swallow | 0.531** | 0.542** | 0.381** | 0.427** | 0.132 | 1 |  |  |  |  |  |
| Breath | 0.557** | 0.546** | 0.404** | 0.314** | 0.123 | 0.627** | 1 |  |  |  |  |
| Oral cavity | 0.202 | 0.126 | 0.259* | 0.138 | 0.154 | 0.204 | 0.098 | 1 |  |  |  |
| Total oral scores | 0.731** | 0.709** | 0.668** | 0.554** | 0.358** | 0.825** | 0.724** | 0.379** | 1 |  |  |
| Texture of cookies | 0.237* | 0.371** | 0.362** | 0.195 | 0.256* | 0.272* | 0.315** | 0.173 | 0.339** | 1 |  |
| Nursing home feeding | 0.348** | 0.456** | 0.282* | 0.339** | 0.338** | 0.422** | 0.325** | 0.153 | 0.470** | 0.495** | 1 |

* *p*<0.05; ***p*<0.01.
